# Mechanobiological Analysis of Molar Teeth with Carious Lesions through the Finite Element Method

**DOI:** 10.1155/2018/1815830

**Published:** 2018-10-14

**Authors:** R. A. Hernández-Vázquez, Betriz Romero-Ángeles, Guillermo Urriolagoitia-Sosa, Juan Alejandro Vázquez-Feijoo, Rodrigo Arturo Marquet-Rivera, Guillermo Urriolagoitia-Calderón

**Affiliations:** Instituto Politécnico Nacional, Escuela Superior de Ingeniería Mecánica y Eléctrica, Sección de Estudios de Posgrado e Investigación, Unidad Profesional Adolfo López Mateos “Zacatenco”, Avenida Instituto Politécnico Nacional s/n, Edificio 5, 2do. Piso, Col. Lindavista, C.P. 07320 Ciudad de Mexico, Mexico

## Abstract

The analysis of the distribution of stress in dental organs is a poorly studied area. That is why computational mechanobiological analysis at the tissue level using the finite element method is very useful to achieve a better understanding of the biomechanics and the behaviour of dental tissues in various pathologies. This knowledge will allow better diagnoses, customize treatment plans, and establish the basis for the development of better restoration materials. In the present work, through the use of high-fidelity biomodels, computational mechanobiological analyses were performed on four molar models affected with four different degrees of caries, which are subjected to masticatory forces. With the analyses performed, it is possible to observe that the masticatory forces that act on the enamel are not transmitted to the dentin and to the bone and periodontal ligament to protect the nerve, as it happens in a healthy dental organ. With the presence of decay, these forces are transmitted partly to the pulp. The reactions to the external loads on the dental organs depend on the advances of the carious lesion that they present, since the distribution of stresses is different in a healthy tooth.

## 1. Introduction

Mechanobiology is an area of recent application in dentistry [1, 2]; this is dedicated to the analysis of stresses and deformations in tissues in living beings. Study tools used in engineering are applied in the structures of living beings. The structures that make up the stomatognathic system have a harmonious morphology with a high degree of specialization. All the bony, neuromuscular, articular, periodontal, and occlusal components establish an equilibrium between them that allow their complex physiology [3]. Even the finest detail, such as a roughness, a groove, a cusp, or an orifice, fulfills a specific function. In particular, when it comes to the chewing function, the collusive components or dental organs are the protagonists. When the total or partial loss of any of these components occurs, there is a loss of the functional balance of the system, which in turn causes the occlusion not only to be altered but also to be lost [4]. This in turn causes alterations in its biomechanics.

**Figure 1:**
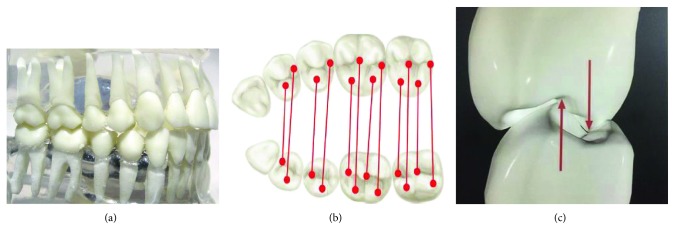
Harmonious relationship of dental morphology. (a) Dental occlusion, (b) points where the upper teeth make contact with the lower teeth, according to their morphology, and (c) detail of occlusal assembly between premolars.

The morphology of each dental organ is designed to perform this function. The occlusal surface of the posterior teeth (molars and premolars) has cusps, depressions, grooves, and pits that coincide with those of the opposing tooth (Figure 1), as well as the incisal edges, cusps, and cingules in the case of the anterior teeth (incisors and canines) [5]. Together with the rest of the components of the system, they allow the teeth to perform their function in a phenomenon called occlusion [6], which is regulated by the masticatory forces acting on each of the teeth.

Due to this, analysis of the distribution of stresses generated by occlusal or masticatory loads, in a biological system such as the teeth, is a complex problem [7], because of the nature of the tissues of the dental organ, such as nonhomogeneous materials and the geometric irregularities of their contours and anatomical forms. In addition to this, the tooth in its structure is formed by enamel, dentine, and pulp, whose mechanical properties differ from one another. The distribution of stresses is also affected by health and pathological states, which makes their analysis even more complex. The teeth concentrates the forces generated by the muscles in small areas such as contact surfaces and cusps or incisal edges.

**Figure 2:**
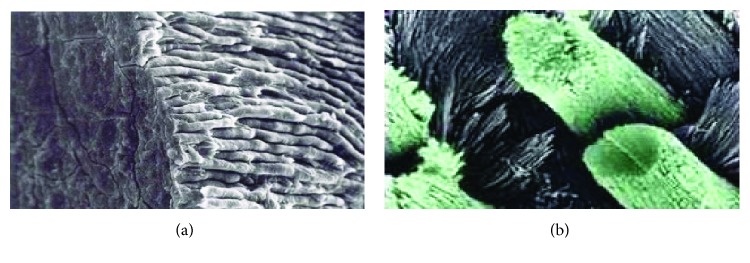
Micrographs of dental enamel. (a) Distribution of enamel prisms and (b) hydroxyapatite prisms.

Dental enamel is the tissue most exposed to high occlusal forces, due to the processing of food during mastication in the oral cavity and the occlusal contact between the teeth, for which it suffers attrition and fractures. It is a highly specialized crystalline structure. The prisms that structure it are package of hydroxyapatite crystals ordered and densely packed by a delicate network of organic material that surrounds them (Figure 2). The prism is considered as the basic structural unit of the enamel, and the prismatic enamel is the set of prisms that constitutes most of the mineralized matrix [8].

For its part, dentine is the one that provides support to enamel and transmits the forces it receives. Its microstructure is dominated by the presence of crystal structures in the shape of an inverted cone called dentinal tubules. Between each tubule, there is a peritubular channel which contains collagen I, hydroxyapatite crystals, and dentinal fluid. It constitutes the organic or intertubular matrix [9].

Sometimes, nature uses processes like those that engineering uses to improve a design; a clear example is what is known as residual stresses, which are used to reinforce materials, with a process called cold hardening. A similar phenomenon occurs in the teeth. It has been found that the mineral particles of the dentin are precompressed, which prevents the propagation of cracks and increases the strength of their biostructure. Dentin is a material similar to bone formed from mineral nanoparticles, mainly hydroxyapatite. These mineral nanoparticles are embedded in collagen fibres, with which they are firmly interconnected. When the collagen fibres contract, the adhered mineral particles get even more compressed. This allows to prevent fissures, and the compression is carried out in such a way that the cracks cannot easily reach the internal part of the teeth, which could damage the pulp [10]. In this case, the residual stresses are represented by the masticatory loads, which propitiate this phenomenon that prevents the propagation of faults, with the simple fact of chewing.

A detailed information on the distribution of stresses and location of the critical load points is of vital importance, since the dental organs have as their main function the adequate transmission of forces to grind the food. Discrete analysis of stress distribution is essential in these organs and tissues, because the main objective is to understand how these efforts are distributed and transmitted and find stress concentrations in specific places due to damage to the tooth as a function of its growth, adaptation, and structural modification [11]. This type of analysis would allow, for example, the control of dental regeneration. Recent studies have documented that there is some regenerative capacity of dental enamel (as when it is subjected to chemical compounds such as fluoride [12]).

**Figure 3:**
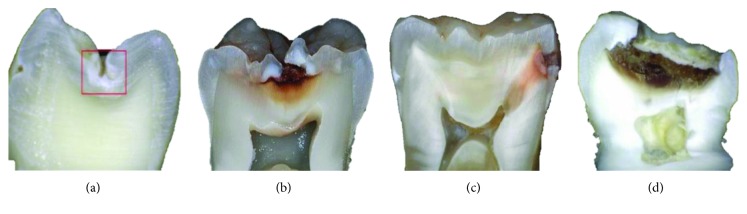
Stages of carious lesions. (a) First stages (enamel), (b) second stages (enamel and dentin), (c) third stages (affectation of the dental nerve), and (d) fourth stages (destruction of morphology).

One of the main pathologies that appear in the teeth is dental caries. This disease, according to the World Health Organization, is a condition and is estimated that five million people worldwide suffer from it [13]. *In global terms, between 60% and 90% of school-age children and nearly 100% of adults have dental caries, often accompanied by pain or feeling of discomfort* [14], being the second most common disease worldwide. It is a chronic multifactorial pathology [15] located in the hard tissues of the dental organ. It is characterized by the loss of the organic matrix due to the action of the organic acid that is produced because of the bacterial metabolism of the microbiota of the oral cavity. It takes place in a progressively way, beginning with the demineralization of the tissue at the ultrastructural level, until it reaches the total loss of the piece [16] (Figure 3).

This condition would cause a total imbalance of the occlusal relationships, since the morphology of the dental organs is altered, up to the loss of the dental organ, which causes the total loss of the interocclusal harmony. In turn, it generates collateral damages such as systemic diseases and residual reactions throughout the stomatognathic System, which is well worth analysing. Therefore, their mechanobiological analysis proposes new guidelines for the actions that can be applied to counteract their affections and to be more certain that the treatments and restorative materials act properly. Likewise, it will establish the foundations of the future of odontology of prevention.

In general, there are no publications that have numerical data on the distribution of efforts as the caries progresses in its different degrees. Therefore, the present work represents a novelty in this subject and field of study. There are some works that raise the possibility of performing analyses of this pathology, but it is not the main objective of them; therefore, they do not present numerical results in relation to the distribution of efforts when a carious lesion occurs. In addition, the models that were made in these studies are theoretical models of reality; they are not models with the characteristics such as the ones we are presenting. The design of the cavities that they propose represents the caries; they are made with cuts to the model, not how the caries is presented in reality.

The biomodels of the present work are the results of radiographic and tomographic images of cases of real caries at different levels, which comply with the natural history of the disease. In addition, no real case is equal to another. They were obtained with a new technique of imaging (cone beam). This system is used to obtain images in difficult to visualize tissues; it is widely used in medicine and dentistry in the craniofacial region. It provides images with a resolution of submillimetres of high diagnostic quality and excellent visualization. In addition to this, the methodology used to generate them ensures biofidelity. For these same reasons, the numerical results would be difficult to compare with other works, given the differences of the models used in other works.

## 2. Materials and Methods

**Figure 4:**
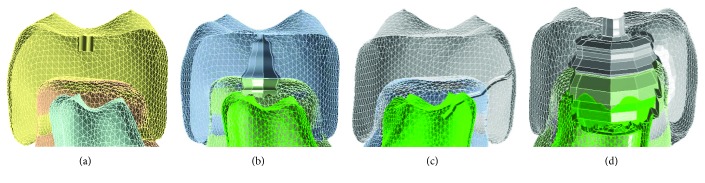
Biomodels with the four degrees of decay, based on (a) first grade, (b) second grade, (c) third grade, and (d) fourth grade.

This study is aimed at carrying out the analysis of four case studies that address the problem of finding the stresses that occur in a dental organ when it is subjected to the forces of occlusion and its structural integrity is compromised, by the presence of carious lesions in its four degrees. Additionally, there is a control case in a healthy molar without decay. For each of the cases, a high-fidelity biomodel of a lower left first molar, representing each stage of caries, was used, obtaining a total of five biomodels represented as follows: control case: without decay; case 1: caries in enamel on the occlusal face, in the main sulcus; case 2: caries in enamel and dentin on the occlusal face, starting in the main sulcus; case 3: caries in enamel and dentin and with pulp communication in the interproximal zone; and case 4: caries of enamel on the occlusal face, in the main sulcus that extends over much of the dentin, causing involvement of the pulp chamber and dental pulp (Figure 4).

For the modelling, the methodology described in a previous work was used [17]. As already mentioned, the images of the molars were obtained through the use of 3D imaging, by means of a digital volumetric tomography (TVD) of the maxilla and mandible with the cone beam computed tomography system (CBTC), to obtain the DICOM files. This system is widely used for studies of the maxillofacial area, since it allows a better visualization of difficult access tissues.

The tomography used is Batex brand model EZ3D, which has a KvP of 90.0 mA (3.8 light beam intensity). A total of 477 images or slices were obtained from each tomography, whose distance between them (slide thickness) is 0.5 mm. The space between the pixels (pixel spacing) is of a ratio of 0.3/0.3 mm. The obtained models have elements of high order (tetrahedral with a total of 10 nodes per element) and consider three different materials that correspond to the tissues that structure the dental organ (enamel, dentine, and pulp).

The FEM analyses were carried out with the ANSYS® computer program. The number of elements and nodes of each biomodel is the following: biomodel of the control case 74,907 elements and 129,005 nodes. First-degree caries have 76,851 elements and 132,043 nodes. Seventy-degree caries have 77,419 elements and 133,497 nodes. Third-degree caries have 80,317 elements and 137,666 nodes. Fourth-degree caries have 81,318 elements and 141,830 nodes.

The structural analysis has an elastic, linear, and homogeneous behaviour. The mechanical properties of the tissues are described in Table 1 [18–20].

The convergences in the generation of discretized meshes were analysed. Discretization was carried out in a semicontrolled way, since the geometries did not allow for a fully controlled discretization, in the same way that an automatic one was not suitable. We chose the size of the Maya that would allow the resolution of the analysis and that the differences between the results would be negligible or consistent.

In relation to the modelling of carious lesions, the following should be mentioned. The axes in tissue ablations of enamel and dentine (caries) are real, where caries is an irregular area, the edges are angular, and therefore the singularities due to the stress concentrators are real and consistent. These irregularities are observed in the images provided in the radiographs and tomographies, so that the resulting biomodels are real reproductions of decayed molars.

**Figure 5:**
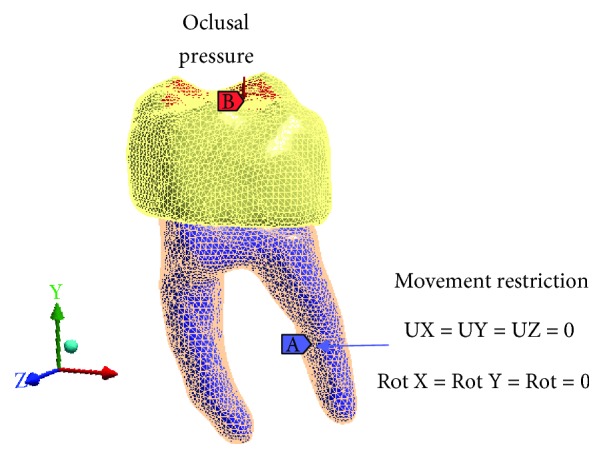
Applied loads and boundary conditions.

The boundary conditions were established in the root zone, restricting the displacements and rotations in the directions of the X, Y, and Z axes. A load was applied on the occlusal surface in the four biomodels. The magnitude of the applied load is 150 N/mm^2^ corresponding to the bite force between the two molars [21–25], which is distributed locally in the application zone on the form of a pressure. So that the pressure is the same in all cases, the area of action is different because of the different degrees of caries they represent. The load is uniform for optimization of the simulation, and this does not affect the results or the conclusions, since it is not a comparison between the cases, so it is not necessary to adjust to the same load (Figure 5).

The contacts between the tissues were considered for the analyses performed. The software used has a function called Contact Manager®, through which the contact simulation was carried out. This tool presents a subfunction called Contact Wizard® in which information was given to establish which material or tissue is the contact and which will be the target. For the simulation, the tissue was established as a contact with the enamel and as a target for the dentin. To establish their contact relationship, a coefficient of friction was also added, which is according to the literature that is established between ceramic materials, since the crystalline nature of these tissues, as well as bone tissue, has been considered as ceramic materials [26].

## 3. Results

Unitary deformation, directional deformation, nominal stresses, shear stresses, principal stresses, and von *Mises stress* were analysed during the application of the load that simulates dental bite or occlusion. It should be mentioned that *Von Mises* stresses are not considered here as a failure criterion (which is mainly applicable to ductile materials), but as a unique nondirectional value that allows to have a global criterion on the load at each tooth point, since it is obtained from the deformation energy. Several authors use it as a criterion to evaluate restorations [27–29].

However, in the body of work, only the efforts of *von Mises* for tissue are shown. This is due to the fact that the main efforts are obviated with those of *von Mises*, since it is the total deformation energy that indicates the magnitude of the stresses. That is, it is representative of the distribution of efforts that is the main objective of this work. In addition, *von Mises* encompasses the shear and normal energy, which can occur in these cases. The shear stresses have the same distribution, and the magnitudes obtained are very similar, and for the objectives of the work, they do not provide more information. In future jobs where the fracture or failure is considered, they could be considered.

The results obtained are shown from Tables 2to 7 and from Figures 6to 20.

**Figure 6:**
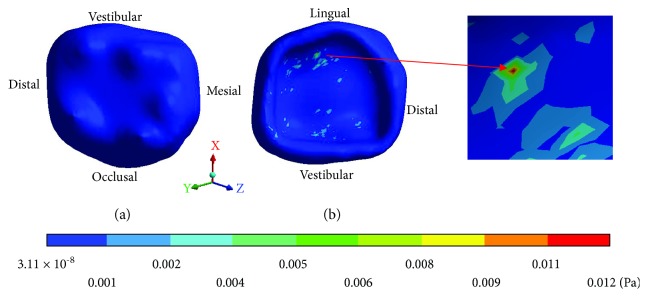
Control case: *von Mises* stresses in enamel: (a) occlusal view (b) cervical view.

**Figure 7:**
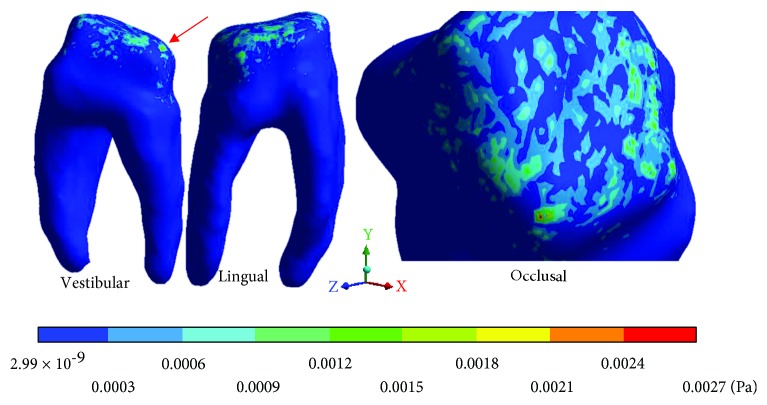
Control case: *von Mises* stresses in dentin.

**Figure 8:**
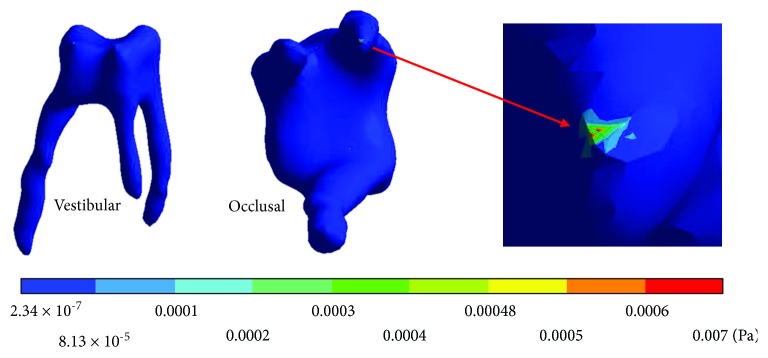
Control case: *von Mises* stresses in pulp.

The *von Mises* stresses of the control case are observed from Figures 6to 8.

**Figure 9:**
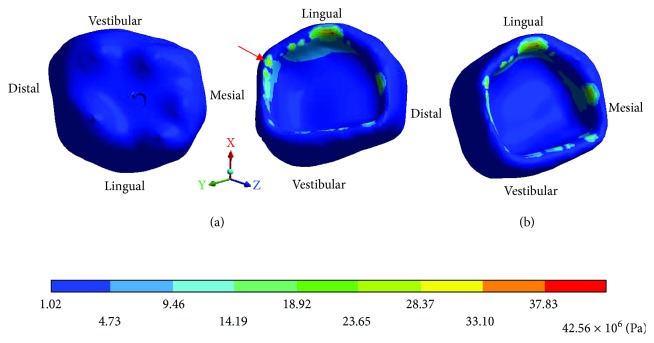
1st degree caries*: von Mises* stresses in enamel: (a) occlusal view (b) cervical view.

**Figure 10:**
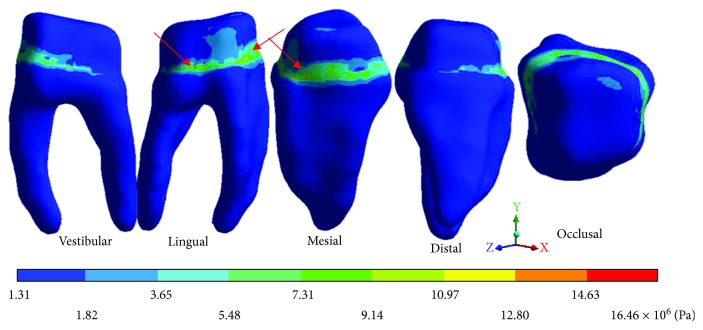
1st degree caries*: von Mises* stresses in dentin.

**Figure 11:**
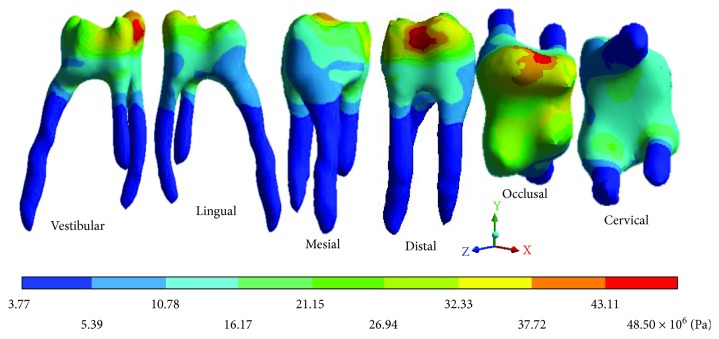
1st degree caries*: von Mises* stresses in pulp.

The *von Mises* stresses of the case 1: 1st degree caries are observed from Figures 9to 11.

**Figure 12:**
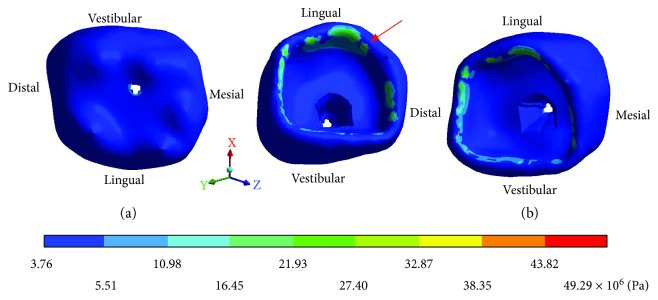
2nd degree caries*: von Mises* stresses in enamel: (a) occlusal view (b) cervical view.

**Figure 13:**
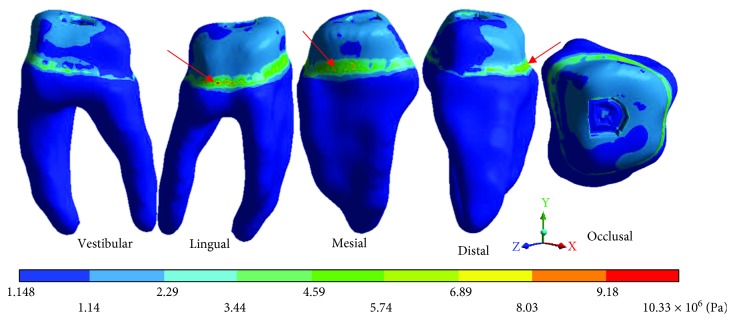
2nd degree caries*: von Mises* stresses in dentin.

**Figure 14:**
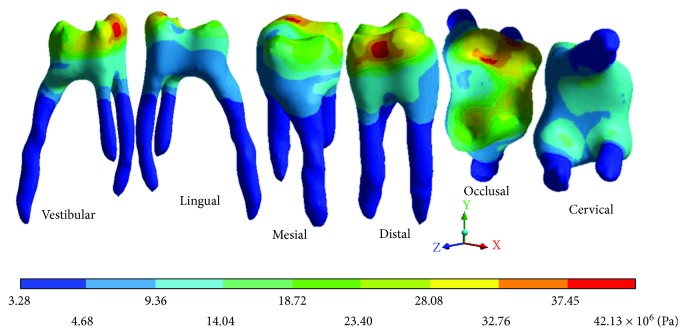
2nd degree caries*: von Mises* stresses in pulp.

The *von Mises* stresses of the case 2: 2nd degree caries are observed from Figures 12to 14.

**Figure 15:**
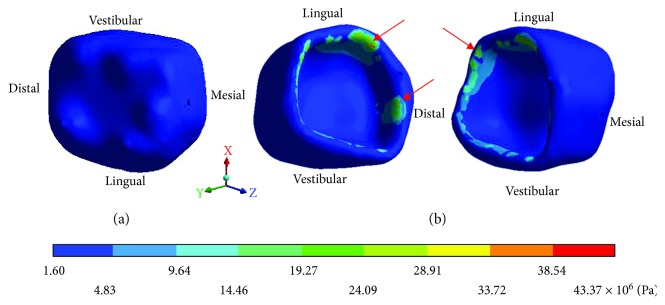
3rd degree caries*: von Mises* stresses in enamel: (a) occlusal view (b) cervical view.

**Figure 16:**
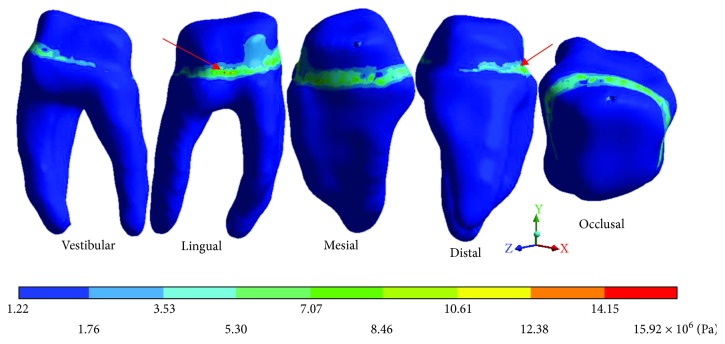
3rd degree caries*: von Mises* stresses in dentin.

**Figure 17:**
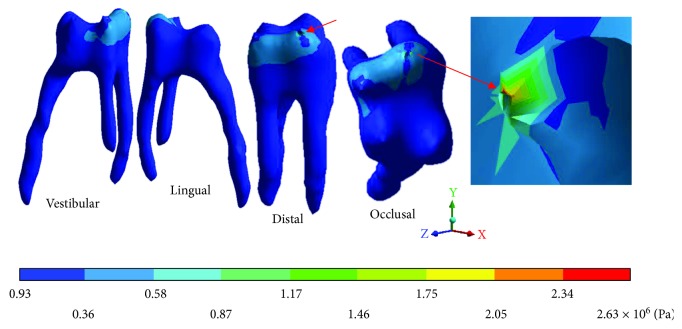
3rd degree caries*: von Mises* stresses in pulp.

The *von Mises* stresses of the case 3: 3rd degree caries are observed from Figures 15to 17.

**Figure 18:**
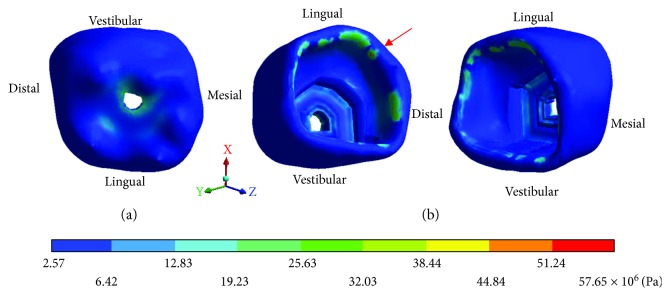
4th degree caries*: von Mises* stresses in enamel: (a) occlusal view (b) cervical view.

**Figure 19:**
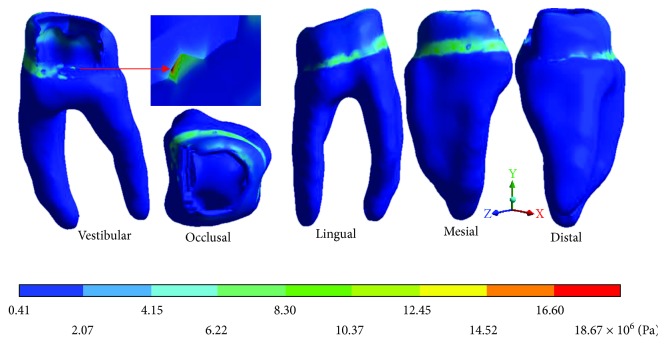
4th degree caries*: von Mises* stresses in dentin.

**Figure 20:**
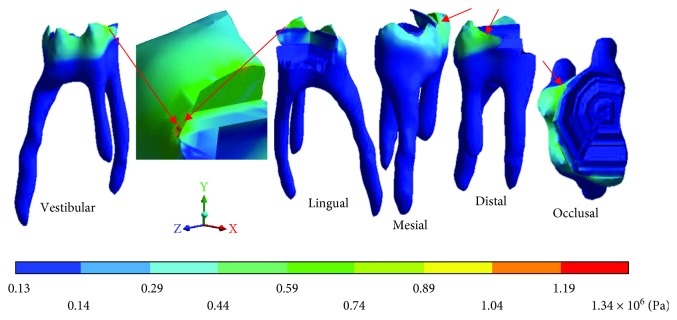
4th degree caries*: von Mises* stresses in pulp.

The *von Mises* stresses of the case 4: 4th degree caries are observed from Figures 18to 20.

## 4. Discussion

With regard to the unitary strain, it can be found that, although the same load is applied in all the study cases, the resulting stress is greater as the degree of caries increases. This would lead to the expectation that greater deformation should occur as the degree of caries increases. However, the less deformation that occurs, the greater the degree of caries. That is, a *stiffening* is generated (to name it somehow) which is due to the interaction of the mechanical properties that are established between the three dental tissues. The rigidity is assumed by the least deformation that was obtained. At this point, it is prudent to clarify that no real case is equal to another; however, aspects such as the pressure exercised are the same in all cases.

The geometric conditions cause that the direct comparison between the different degrees of decay can give the idea that it is due to these differences in the geometry, the motive of the results, and the nature of the unit deformations. However, the effect of stiffening in all four models is consistent. In addition, to represent what actually happens with the natural history of caries disease, the biomodels used are not a continuous growth of the same caries and are not an elaborate design to control, but are results of intakes radiographic and tomographic of cases of real caries at different levels, which comply with the natural history of the disease, which is why many aspects are not directly comparable.

This could suggest that the number of variables that can be presented would compromise the results of the analyses. However, such control would distance work from reality. As already mentioned, it is not the intention to compare one case with another since; to make a comparison, it would have to grow the same decay. Each caries is different, and therefore, the efforts are not comparable. What is tried to demonstrate is that, in all cases, there are first efforts in the area of the amelodentinaria union and that, as the degree of caries increases, the pulp begins to load, unlike the control case where the dental organ is healthy and the pulp is not reached by any kind of effort.

At the moment that the caries appears, begin to present efforts. It is possible to come to think that in a controlled and designed case that is uniform, the pulp can come to present greater load (it is not possible to do this in a real case because it would have to be done in a healthy molar and that is allowed to get sick with caries, without getting to treat it until it reaches a fourth grade, which is ethically complicated that can be done). So, to achieve this, it would be necessary to develop a theoretical model away from reality.

It is also true that, in the present work, the pulp is modelled as a complete tissue, when in reality it is a vascular-nervous package. However, this condition is consistent in many publications that do so, since it is a very appropriate approximation model. It is possible to consider that this behaviour could only be proven in an experimental model, which would also be complicated, since the blood vessels and nerves that make it up are capillary. But there are still no conditions to be able to perform in this way. Nevertheless, as already mentioned, this does not change the way in which the effort is being distributed. This makes the biomodels more than adequate. This does not mean that, in future investigations, optimizations or anisotropic or even orthotropic analyses cannot be made.

Specifically, with the *von Mises* stresses, it is possible to observe that the values in comparison with the control case are increased. In the same way, this is related to the structural integrity of the molar for each case of caries; the geometry modified by the degree of caries is a factor that affects these results, due to structural change. The tissue loss varies in each case. In addition, the occlusal forces that act (horizontal and vertical) play an important role in these results, since the disposition and depth of the lesion modify the resulting efforts and reactions.

This could again suggest the idea that it is due to the way in which caries was generated in biomodels that singularities are presented as stress concentrators and intensifiers. In clinical cases, this is a reality. Caries produces irregularities in the geometry of dental morphology. These irregularities actually produce stress concentrators; omitting them in the generation of the biomodel would modify the results of what actually occurs in the molar with decay.

## 5. Conclusions

With the present work, it was possible to demonstrate that the presence of caries causes mainly three phenomena:

1. There is an overload in the contact of the amelodentinaria junction.
2. This demonstrates the traditional empirical knowledge of dentistry that the dental organ can fail due to the loss of elasticity provided by dentin.
3. The simple fact of presenting a carious lesion causes the pulp to be affected from a mechanical point of view.

In an investigation published prior to the present work [17], it was found that there are stress concentrations in the amelodentinous junction (located in the anatomical neck of the teeth, it is the junction or articulation zone of enamel and dentin) in healthy molars due to the simple fact of mastication. Not only does this work corroborate this fact, but also when the dental organ presents caries, one of the first consequences is that in the amelodentinous junction greater points of concentration of efforts are manifested. Establishing with this are areas with propensity to present lesions known as noncarious, before functional loads and not for functional loads, as it usually happens as a precedent of this type of injuries.

On the other hand, when the caries causes the dentin to decrease, the elasticity of the dental organ tissue system is lost as well, since this property is provided by this tissue. Therefore, there is a predisposition to failure, mainly the enamel, which is consistent with the existing empirical knowledge in dentistry, which states that a dental organ that suffers from caries has a greater risk of fracture by force of normal bite, than a healthy tooth. It is common to hear from dentists that the simple fact of presenting decay makes the tooth more fragile and the greater the degree of decay, the greater the risk of that organ ruptures.

With this, it is possible to establish that, in addition to biological and chemical factors that cause that there is greater predisposition to the failure of a dental organ with caries, there are factors of a mechanical nature that cause this failure. In this same way, not only are biological and chemical factors cause irritation in the pulp that in the end will trigger inflammation, pain, and even necrosis. When losing the dentine or seeing its elastic property diminished, its function of dissipating the masticatory forces decreases, with which the pulp is involved in the distribution of stresses that do not occur in a healthy tooth.

With this it can be deduced that regardless of the size or depth of the caries, the pulp will be affected as long as the dentin loses its elastic function. This should cause reflection that, once the decay is eliminated and the materials to restore the remaining tissues are selected, they must restore the lost elastic functions of the system, in such a way that the pula is no longer involved and it is possible to reestablish the health and functioning of the dental organ and its tissues.

Therefore, the present work serves to establish the importance of seeing caries from another point of view, from a biomechanical point of view, and the cellular responses that can be generated (mechanobiology and mechanotransduction). It allows considering mechanical stresses and efforts as agents that also participate in the injury of dental tissues affected with caries. With this, focus the bases for the generation of new materials or even biointelligent materials, i.e., materials that mimic the behaviour of biological tissues. In this case, the results obtained show that the function of dental tissues, specifically dentin, can be modified due to the presence of external factors such as pathology. Caries not only modifies the biological and chemical structure of dental tissues; it also alters its mechanical function.

In the future, the materials that should be used will depend to a great extent on whether they are capable of reproducing the natural function of the original tissues and that they are also capable of solving the changes that were caused by the presence of caries. For this, it is necessary that these materials mimic the biological tissues and by themselves offer means of rehabilitation rather than substitution. As already mentioned, there are studies that affirm that the biological tissues when stimulated mechanically accelerate their healing and remodelling, managing to accelerate their recovery. It has been found that, as already mentioned, as the mechanical stimulation of the dentine produces its regeneration, then it would be desirable that the restorative materials could fulfill that function of stimulation that would lead to the regeneration of the damaged tissue.

## Figures and Tables

**Table 1:** Mechanical properties used in the analysis.

	Enamel	Dentin	Pulp
Density	0.25 g/cm^3^	0.31 g/cm^3^	0.1 g/cm^3^
*Young's module*	70 GPa	18.3 GPa	2 GPa
Dimensional *Poisson* ratio	0.30	0.30	0.45

**Table 2:** Unitary strain.

Control case	1st degree caries	2nd degree caries	3rd degree caries	4th degree caries
3.66 × 10^−5^	3.06 × 10^−12^	3.80 × 10^−16^	5.04 × 10^−16^	6.07 × 10^−16^

**Table 3:** Control case: *von Mises* stresses results.

Values	Enamel	Dentin	Pulp
Maximum	0.012 Pa	0.0027 Pa	0.0007
Minimum	3.11 × 10^−8^ Pa	2.99 × 10^−9^ Pa	2.34 × 10^−7^ Pa

**Table 4:** 1st degree caries*: von Mises* stresses results.

Values	Enamel	Dentin	Pulp
Maximum	1.42 × 10^6^ Pa	16.46 × 10^6^ Pa	48.50 × 10^6^ Pa
Minimum	1.02 × 10^6^ Pa	1.31 × 10^6^ Pa	3.77 × 10^6^ Pa

**Table 5:** 2nd degree caries*: von Mises* stresses results.

Values	Enamel	Dentin	Pulp
Maximum	49.29 × 10^6^ Pa	10.33 × 10^6^ Pa	42.13 × 10^6^ Pa
Minimum	3.76 × 10^6^ Pa	1.14 × 10^6^ Pa	3.28 × 10^6^ Pa

**Table 6:** 3rd degree caries*: von Mises* stresses results.

Values	Enamel	Dentin	Pulp
Maximum	43.37 × 10^6^ Pa	15.92 × 10^6^ Pa	2.63 × 10^6^ Pa
Minimum	1.60 × 10^6^ Pa	1.22 × 10^6^ Pa	0.93 × 10^6^ Pa

**Table 7:** 4th degree caries*: von Mises* stresses results.

Values	Enamel	Dentin	Pulp
Maximum	57.65 × 10^6^ Pa	18.67 × 10^6^ Pa	1.34 × 10^6^ Pa
Minimum	2.57 × 10^6^ Pa	0.41 × 10^6^ Pa	0.13 × 10^6^ Pa

## Data Availability

The data used to support the findings of this study are included within the article.
